# Supplementary material for: Factors Correlated with Body Image Dissatisfaction in Children and Adolescents Diagnosed with HIV: A Cross-Sectional Study
Source: Int J Environ Res Public Health. 2022 Oct 30;19(21):14197. doi: 10.3390/ijerph192114197 (PMC9654128; doi:10.3390/ijerph192114197)
Supplement: Supplementary file 1 [file ijerph-19-14197-s001.zip › ijerph-1908973-supplementary.pdf]

SUPPLEMENTARY FILE

Table S1. Comparison of current silhouette, ideal silhouette and body image score according to independent variables in female children and adolescents diagnosed with HIV infection (n = 32). Florianópolis – SC, 2015/2016.

	**Current silhouette Mean (sd)**	**p-value**	**Ideal silhouette Mean (sd)**	**p-value**	**Body image score Mean (sd)**	**p-value**
**Age** §		0.895		0.873		0.681
8-10	2.00 (1.41)		1.85 (1.57)		0.14 (0.89)	
11-15	1.92 (1.28)		1.96 (0.97)		-0.04 (1.36)	
**Sexual maturation** §		0.930		0.512		0.205
Prepubescent	2.00 (1.73)		2.40 (1.67)		-0.40 (0.54)	
Pubescent	1.92 (1.23)		1.85 (0.98)		0.07 (1.35)	
**Economic Level** §		0.149		0.771		0.172
High purchasing power	2.40 (0.54)		1.80 (1.09)		0.60 (0.89)	
Low purchasing power	1.85 (1.37)		1.96 (1.12)		-0.11 (1.31)	
**ART use** §		0.775		0.751		0.809
Yes	1.89 (1.50)		1.93 (1.16)		-0.03 (1.17)	
No	2.33 (2.30)		2.00 (0.00)		0.33 (2.30)	
**Physical activity** §		0.877		0.07		0.270
Physically Active	1.85 (1.57)		1.28 (0.95)		0.57 (1.51)	
Little Physically Active	1.96 (1.24)		2.12 (1.09)		-0.16 (1.17)	
**Television Time (week)** §		0.492		0.722		0.538
≥ 2 hours	2.60 (2.30)		2.20 (1.78)		0.40 (1.51)	
<2 hours	1.81 (1.04)		1.88 (0.97)		-0.07 (1.23)	
**Television time (weekend)**§		0.587		0.341		0.780
≥ 2 hours	1.80 (1.47)		2.11 (0.99)		-0.05 (1.47)	
<2 hours	2.05 (1.14)		1.73 (1.22)		0.06 (1.03)	
**Computer and Video Game Time (week)** §		0.661		0.846		0.796
≥ 2 hours	1.75 (1.38)		1.95 (1.16)		-0.12 (1.64)	

<2 hours	2.00 (1.28)		1.87 (0.99)		0.04 (1.16)	
**Computer and Video Game Time (weekend)** §		**0.015***		0.904		0.188
≥ 2 hours	1.20 (0.44)		2.00 (1.22)		-0.80 (1.30)	
<2 hours	2.07 (1.35)		1.92 (1.10)		0.14 (1.23)	
**Lipoatrophy**§		0.866		0.624		0.430
Present	2.00 (1.00)		1.71 (1.38)		0.285 (0.95)	
Absent	1.92 (1.38)		2.00 (1.04)		-0.08 (1.35)	
**Body mass index** †		0.813		**0.007***		0.057
Eutrophy	1.92 (1.22)		1.64 (0.90)		0.28 (1.17)	
Thinness	1.50 (2.12)		2.50 (0.70)		-1.00 (1.41)	
Overweight	2.20 (1.64)		3.20 (1.30)		-1.00 (1.22)	

≥: menor ou igual; <: maior; p: p-value; †: One-way Anova test; §: Student t test for independent samples; *:p<0.05, sd: standard deviation.

Table S2. Comparison of current silhouette, ideal silhouette and body image score according to independent variables in male children and adolescents diagnosed with HIV infection (n = 28). Florianópolis – SC, 2015/2016.

	Current silhouette Mean (sd)	p-value	Ideal silhouette Mean (sd)	p-value	Body image score Mean (sd)	p-value
**Age** §		0.917		0.116		0,205
8-10	2.42 (0.97)		1.57 (1.27)		0.85 (1.77)	
11-15	2.38 (1.20)		2.52 (1.28)		-0.14(1.31)	
**Sexual maturation** §		0.789		0.062		0,119
Prepubescent	2.50 (1.04)		1.33 (1.21)		1.16 (1.72)	
Pubescent	2.36 (1.17)		2.54 (1.26)		-0.18 (1.29)	
**Economic Level** §		0.077		0.266		0,704
High purchasing power	2.00 (0.00)		2.00 (0.00)		0.00 (0.00)	
Low purchasing power	2.42 (1.17)		2.30 (1.37)		0.11 (1.53)	
**ART use** §		0.561		0.056		0,171
Yes	2.33 (1.23)		2.00 (1.26)		-0.57 (1.39)	
No	2.57 (0.78)		3.14 (1.21)		0.33 (1.46)	
**Physical activity** §		0.914		0.585		0,566
Physically Active	2.36 (1.12)		2.45 (1.21)		-0.09 (1.37)	
Little Physically Active	2.41 (1.17)		2.17 (1.42)		0.23 (1.56)	
**Television Time (week)** §		0.127		0.863		0,208
≥ 2 hours	1.88 (1.16)		2.22 (1.30)		-0.33 (1.00)	
<2 hours	2.63 (1.06)		2.31 (1.37)		0.31 (1.63)	
**Television time (weekend)**§		1.107		0.458		0,602
≥ 2 hours	2.66 (1.02)		2.00 (1.63)		0.22 (1.43)	
<2 hours	1.90 (1.19)		2.44 (1.14)		-0.10 (1.59)	
**Computer and Video Game Time (week)** §		0.971		0.229		0,276
≥ 2 hours	2.38 (0.96)		2.61 (1.32)		-0.23 (1.64)	
<2 hours	2.40 (1.29)		2.00 (1.30)		0.40 (1.29)	
**Computer and Video Game Time**		0.190		0.232		0,922

**(weekend)** §						
≥ 2 hours	2.69 (0.94)		2.61 (1.38)		0.07 (1.55)	
<2 hours	2.13 (1.24)		2.00 (1.25)		0.13 (1.45)	
**Lipoatrophy**§						
Present						
Absent	2.39 (1.13)		2.29 (1.32)		0.11 (1.47)	
**Body mass index** †		0.394		0.930		0,627
Eutrophy	2.54 (1.01)		2.31 (1.32)		0.22 (1.60)	
Thinness	1.66 (2.08)		2.33 (2.08)		-0.66 (1.15)	
Overweight	2.00 (1.00)		2.00 (1.00)		0.00 (0.00)	

≥: menor ou igual; <: maior; p: p-value; †: One-way Anova test; §: Student t test for independent samples; *:p<0.05, sd: standard deviation.

Table S3. Pearson and Spearman correlation between body image score and independent variables of the study in children and adolescents diagnosed with HIV infection (n=60). Florianópolis – SC, 2015/2016.

	**Total Sample** (n=60)		**Women** (n=32)		**Male** (n = 28)	
	**r**	**p**	**r**	**p**	**r**	**p**
**Sex†**	0.062	0.635	-		-	
**Age**	-0.210	0.107	0.065	0.722	-0.462	**0.013**
**Sexual maturation †**	-0.073	0.575	0.153	0.400	-0.296	0.126
**Economic Level †**	-0.149	0.254	-0.278	0.123	0.018	0.927
**ART†**	-0.114	0.381	-0.062	0.736	-0.205	0.295
**CD4 lymphocytes (cells.mm-³)**	0.054	0.681	0.066	0.719	0.045	0.821
**CD8 lymphocytes (cells.mm-³)**	-0.093	0.477	-0.095	0.605	-0.092	0.641
**Viral Load (log)**	-0.168	0.200	-0.017	0.930	-0.338	0.079
**Physical activity †**	-0.035	0.787	-0.187	0.304	0.134	0.497
**Television Time (week)†**	-0.037	0.776	0.094	0.607	-0.180	0.359
**Television time (weekend) †**	0.009	0.943	-0.058	0.753	0.073	0.711
**Computer and Video Game Time (week) †**	-0.125	0.341	-0.024	0.892	-0.234	0.230
**Computador e Computer and Video Game Time (weekend) †**	-0.053	0.685	-0.209	0.252	0.023	0.906
**Lipoatrophy †**	-0.084	0.506	-0.170	0.352	-	-
**BMI**	-0.084	0.525	0.023	0.902	-0.207	0.290
**Body fat (%)**	-0.045	0.734	-0.111	0.542	0.007	0.970
**TSF (mm)**	-0.043	0.744	-0.031	0.865	-0.063	0.751
**SSF (mm)**	-0.086	0.512	-0.138	0.451	-0.013	0.949
**ASF (mm)**	-0.029	0.824	0.050	0.786	-0.162	0.411
**CSF (mm)**	0.063	0.630	0.065	0.725	0.073	0.713
**Self-esteem Score**	-0.102	0.438	-0.123	0.502	-0.083	0.675

†: Spearman's Correlation, r: correlation coefficient; %: percentage, ≥: menor ou igual; <: maior; BMI: body mass index; Sex: 1 = Female; 2 = Male; Age: 0=8 a 10 years; 1=11 a 15 anos; sexual maturation: 0= Prepubescent; 1= Pubescent; Economic Level: 0= High purchasing power; 1= Low purchasing power; ART use:1= yes; 0= noo; Physical activity: 0= Physically Active; 1= Little Physically Active; Television Time (week): 0= ≥2 hours; 1= <2 hours; Television time (weekend): 0= ≥2 hours; 1= <2 hours; Computer and Video Game Time (week): 0= ≥2 hours; 1= <2 hours; Computer and Video Game Time (weekend): 0= ≥2 hours; 1= <2 hours; Lipoatrofia: 1= presente; 0=ausente, TSF: triceps skinfold; SSF: subscapular skinfold; ASF: abdominal skinfold, CSF: calf skinfold
